# Supplementary material for: Multi‐dataset identification of innovative feature genes and molecular mechanisms in keratoconus
Source: J Cell Mol Med. 2024 Sep 19;28(18):e70079. doi: 10.1111/jcmm.70079 (PMC11412914; doi:10.1111/jcmm.70079)
Supplement: Supplementary file 2 — Material S2. [file JCMM-28-e70079-s002.zip › 7.GSEA analysis/3.DBNDD1/KEGG_PATHWAYS_IN_CANCER.html]

Details for gene set KEGG\_PATHWAYS\_IN\_CANCER[GSEA]

|  || Dataset | DBNDD1.group.cls#h\_versus\_l |
| Phenotype | group.cls#h\_versus\_l |
| Upregulated in class | l |
| GeneSet | KEGG\_PATHWAYS\_IN\_CANCER |
| Enrichment Score (ES) | -0.29342917 |
| Normalized Enrichment Score (NES) | -1.2689083 |
| Nominal p-value | 0.15127702 |
| FDR q-value | 0.6490085 |
| FWER p-Value | 0.974 |
Table: GSEA Results Summary

  

Fig 1: Enrichment plot: KEGG\_PATHWAYS\_IN\_CANCER      
 Profile of the Running ES Score & Positions of GeneSet Members on the Rank Ordered List

  

| SYMBOL | TITLE | RANK IN GENE LIST | RANK METRIC SCORE | RUNNING ES | CORE ENRICHMENT || 1 | PIK3R5 | na | 35 | 0.708 | 0.0202 | No |
| 2 | FGF16 | na | 48 | 0.676 | 0.0402 | No |
| 3 | FGF14 | na | 999 | 0.383 | 0.0241 | No |
| 4 | DCC | na | 1372 | 0.347 | 0.0237 | No |
| 5 | RAC3 | na | 1824 | 0.312 | 0.0200 | No |
| 6 | MECOM | na | 1978 | 0.301 | 0.0246 | No |
| 7 | FGF8 | na | 2224 | 0.286 | 0.0261 | No |
| 8 | FGF17 | na | 2525 | 0.271 | 0.0255 | No |
| 9 | RARB | na | 2894 | 0.256 | 0.0225 | No |
| 10 | FGF23 | na | 3173 | 0.245 | 0.0217 | No |
| 11 | PDGFRA | na | 3213 | 0.243 | 0.0279 | No |
| 12 | HGF | na | 3455 | 0.233 | 0.0279 | No |
| 13 | CBLB | na | 4045 | 0.212 | 0.0172 | No |
| 14 | TGFBR2 | na | 4515 | 0.197 | 0.0095 | No |
| 15 | FLT3 | na | 4749 | 0.188 | 0.0083 | No |
| 16 | CCNE2 | na | 4812 | 0.186 | 0.0121 | No |
| 17 | FGF21 | na | 5114 | 0.176 | 0.0087 | No |
| 18 | RUNX1T1 | na | 5581 | 0.162 | -0.0000 | No |
| 19 | LAMB4 | na | 5688 | 0.159 | 0.0017 | No |
| 20 | TRAF3 | na | 5973 | 0.151 | -0.0020 | No |
| 21 | PRKCG | na | 6020 | 0.150 | 0.0011 | No |
| 22 | AR | na | 6232 | 0.144 | -0.0007 | No |
| 23 | E2F2 | na | 6645 | 0.133 | -0.0086 | No |
| 24 | FGF18 | na | 6694 | 0.132 | -0.0061 | No |
| 25 | WNT16 | na | 6810 | 0.129 | -0.0056 | No |
| 26 | PIK3R1 | na | 6912 | 0.126 | -0.0047 | No |
| 27 | CCNA1 | na | 7073 | 0.123 | -0.0057 | No |
| 28 | SMAD2 | na | 7383 | 0.116 | -0.0112 | No |
| 29 | LAMC3 | na | 7702 | 0.110 | -0.0171 | No |
| 30 | MMP2 | na | 7845 | 0.107 | -0.0180 | No |
| 31 | IGF1R | na | 8164 | 0.100 | -0.0242 | No |
| 32 | PTCH2 | na | 8169 | 0.100 | -0.0213 | No |
| 33 | SKP2 | na | 8381 | 0.097 | -0.0246 | No |
| 34 | TRAF5 | na | 8439 | 0.096 | -0.0234 | No |
| 35 | CEBPA | na | 8562 | 0.093 | -0.0241 | No |
| 36 | SOS1 | na | 8586 | 0.093 | -0.0220 | No |
| 37 | FGF9 | na | 8610 | 0.092 | -0.0199 | No |
| 38 | FASLG | na | 8688 | 0.091 | -0.0194 | No |
| 39 | FGF12 | na | 8755 | 0.090 | -0.0186 | No |
| 40 | SPI1 | na | 9071 | 0.084 | -0.0252 | No |
| 41 | CREBBP | na | 9242 | 0.082 | -0.0277 | No |
| 42 | FZD5 | na | 9252 | 0.082 | -0.0255 | No |
| 43 | PDGFA | na | 9340 | 0.081 | -0.0256 | No |
| 44 | AKT2 | na | 9388 | 0.080 | -0.0246 | No |
| 45 | CASP8 | na | 9432 | 0.079 | -0.0234 | No |
| 46 | LAMA2 | na | 9618 | 0.077 | -0.0265 | No |
| 47 | CCND1 | na | 9676 | 0.076 | -0.0259 | No |
| 48 | FZD3 | na | 9718 | 0.075 | -0.0248 | No |
| 49 | FZD4 | na | 9743 | 0.075 | -0.0233 | No |
| 50 | MDM2 | na | 9756 | 0.075 | -0.0214 | No |
| 51 | MAPK3 | na | 9865 | 0.073 | -0.0223 | No |
| 52 | WNT9A | na | 9937 | 0.072 | -0.0222 | No |
| 53 | PDGFRB | na | 10046 | 0.071 | -0.0232 | No |
| 54 | NTRK1 | na | 10107 | 0.070 | -0.0229 | No |
| 55 | RB1 | na | 10147 | 0.069 | -0.0219 | No |
| 56 | CTBP1 | na | 10322 | 0.067 | -0.0249 | No |
| 57 | BCR | na | 10482 | 0.065 | -0.0276 | No |
| 58 | FZD1 | na | 10494 | 0.065 | -0.0260 | No |
| 59 | MAPK10 | na | 10497 | 0.065 | -0.0241 | No |
| 60 | IGF1 | na | 10555 | 0.064 | -0.0239 | No |
| 61 | WNT8A | na | 10620 | 0.063 | -0.0238 | No |
| 62 | FADD | na | 10784 | 0.061 | -0.0267 | No |
| 63 | MAP2K2 | na | 10931 | 0.059 | -0.0292 | No |
| 64 | PTEN | na | 10959 | 0.059 | -0.0282 | No |
| 65 | APPL1 | na | 10995 | 0.058 | -0.0275 | No |
| 66 | BID | na | 11063 | 0.057 | -0.0277 | No |
| 67 | COL4A1 | na | 11293 | 0.055 | -0.0327 | No |
| 68 | FGF5 | na | 11314 | 0.054 | -0.0317 | No |
| 69 | LAMA5 | na | 11354 | 0.054 | -0.0312 | No |
| 70 | MAPK9 | na | 11537 | 0.052 | -0.0349 | No |
| 71 | PIK3R2 | na | 11699 | 0.050 | -0.0381 | No |
| 72 | CTNNA2 | na | 11866 | 0.048 | -0.0415 | No |
| 73 | PLCG1 | na | 11955 | 0.048 | -0.0426 | No |
| 74 | SMAD4 | na | 11961 | 0.047 | -0.0413 | No |
| 75 | RET | na | 11988 | 0.047 | -0.0407 | No |
| 76 | RXRA | na | 12017 | 0.047 | -0.0401 | No |
| 77 | FGF22 | na | 12142 | 0.046 | -0.0423 | No |
| 78 | XIAP | na | 12176 | 0.045 | -0.0419 | No |
| 79 | FGFR3 | na | 12198 | 0.045 | -0.0411 | No |
| 80 | CBL | na | 12212 | 0.045 | -0.0402 | No |
| 81 | MAX | na | 12590 | 0.042 | -0.0499 | No |
| 82 | FGF2 | na | 13023 | 0.038 | -0.0613 | No |
| 83 | MTOR | na | 13075 | 0.038 | -0.0616 | No |
| 84 | ERBB2 | na | 13132 | 0.037 | -0.0621 | No |
| 85 | CASP3 | na | 13339 | 0.035 | -0.0671 | No |
| 86 | SUFU | na | 13357 | 0.035 | -0.0665 | No |
| 87 | FOS | na | 13400 | 0.035 | -0.0667 | No |
| 88 | FZD8 | na | 13456 | 0.035 | -0.0672 | No |
| 89 | BAX | na | 13486 | 0.034 | -0.0670 | No |
| 90 | RBX1 | na | 13500 | 0.034 | -0.0664 | No |
| 91 | SOS2 | na | 13580 | 0.033 | -0.0677 | No |
| 92 | TGFB3 | na | 13624 | 0.033 | -0.0679 | No |
| 93 | DAPK1 | na | 13939 | 0.030 | -0.0762 | No |
| 94 | TP53 | na | 13962 | 0.030 | -0.0759 | No |
| 95 | WNT10B | na | 14038 | 0.030 | -0.0772 | No |
| 96 | KRAS | na | 14127 | 0.029 | -0.0789 | No |
| 97 | MSH3 | na | 14141 | 0.029 | -0.0784 | No |
| 98 | PIK3R3 | na | 14144 | 0.029 | -0.0776 | No |
| 99 | HSP90AA1 | na | 14230 | 0.028 | -0.0792 | No |
| 100 | WNT3A | na | 14444 | 0.027 | -0.0846 | No |
| 101 | FGF3 | na | 14453 | 0.027 | -0.0840 | No |
| 102 | HDAC1 | na | 14495 | 0.026 | -0.0844 | No |
| 103 | PTCH1 | na | 14497 | 0.026 | -0.0836 | No |
| 104 | FZD6 | na | 14642 | 0.025 | -0.0871 | No |
| 105 | RALGDS | na | 14647 | 0.025 | -0.0864 | No |
| 106 | HHIP | na | 14730 | 0.025 | -0.0880 | No |
| 107 | RAC1 | na | 14759 | 0.025 | -0.0881 | No |
| 108 | FN1 | na | 14969 | 0.023 | -0.0935 | No |
| 109 | RALA | na | 15036 | 0.023 | -0.0947 | No |
| 110 | PLD1 | na | 15421 | 0.020 | -0.1053 | No |
| 111 | CBLC | na | 15442 | 0.020 | -0.1053 | No |
| 112 | BRAF | na | 15471 | 0.019 | -0.1055 | No |
| 113 | FOXO1 | na | 15621 | 0.018 | -0.1093 | No |
| 114 | FGF20 | na | 15746 | 0.018 | -0.1124 | No |
| 115 | CTBP2 | na | 15747 | 0.018 | -0.1119 | No |
| 116 | FLT3LG | na | 15748 | 0.018 | -0.1113 | No |
| 117 | STAT5B | na | 16077 | 0.015 | -0.1204 | No |
| 118 | FH | na | 16255 | 0.014 | -0.1251 | No |
| 119 | RASSF5 | na | 16344 | 0.014 | -0.1273 | No |
| 120 | NCOA4 | na | 16364 | 0.013 | -0.1274 | No |
| 121 | CTNNB1 | na | 16378 | 0.013 | -0.1274 | No |
| 122 | ITGA2B | na | 16436 | 0.013 | -0.1287 | No |
| 123 | VHL | na | 16864 | 0.010 | -0.1408 | No |
| 124 | TRAF6 | na | 17024 | 0.009 | -0.1451 | No |
| 125 | PIK3CA | na | 17073 | 0.009 | -0.1462 | No |
| 126 | WNT5A | na | 17212 | 0.008 | -0.1500 | No |
| 127 | RALBP1 | na | 17251 | 0.008 | -0.1509 | No |
| 128 | CCNE1 | na | 17317 | 0.007 | -0.1525 | No |
| 129 | GSTP1 | na | 17472 | 0.006 | -0.1568 | No |
| 130 | VEGFB | na | 17569 | 0.006 | -0.1594 | No |
| 131 | FGF10 | na | 17845 | 0.004 | -0.1673 | No |
| 132 | TRAF4 | na | 17861 | 0.004 | -0.1676 | No |
| 133 | FGF19 | na | 17912 | 0.003 | -0.1690 | No |
| 134 | FGF7 | na | 18036 | 0.002 | -0.1725 | No |
| 135 | FGF13 | na | 18153 | 0.002 | -0.1758 | No |
| 136 | CTNNA1 | na | 18276 | 0.001 | -0.1793 | No |
| 137 | CDH1 | na | 18305 | 0.001 | -0.1801 | No |
| 138 | PIK3CB | na | 18338 | 0.000 | -0.1811 | No |
| 139 | STK4 | na | 18367 | 0.000 | -0.1819 | No |
| 140 | PIAS1 | na | 18429 | -0.000 | -0.1836 | No |
| 141 | LAMA4 | na | 18632 | -0.002 | -0.1894 | No |
| 142 | CHUK | na | 18953 | -0.004 | -0.1986 | No |
| 143 | HRAS | na | 18984 | -0.004 | -0.1994 | No |
| 144 | RAF1 | na | 19005 | -0.005 | -0.1998 | No |
| 145 | AKT1 | na | 19066 | -0.005 | -0.2014 | No |
| 146 | RHOA | na | 19096 | -0.005 | -0.2021 | No |
| 147 | CUL2 | na | 19122 | -0.005 | -0.2026 | No |
| 148 | ARNT | na | 19123 | -0.005 | -0.2025 | No |
| 149 | TGFB2 | na | 19303 | -0.007 | -0.2075 | No |
| 150 | EGF | na | 19333 | -0.007 | -0.2081 | No |
| 151 | PLCG2 | na | 19340 | -0.007 | -0.2081 | No |
| 152 | FGF6 | na | 19523 | -0.009 | -0.2131 | No |
| 153 | AXIN1 | na | 19524 | -0.009 | -0.2128 | No |
| 154 | KIT | na | 19563 | -0.009 | -0.2137 | No |
| 155 | JUP | na | 19747 | -0.011 | -0.2186 | No |
| 156 | BIRC2 | na | 19822 | -0.011 | -0.2205 | No |
| 157 | MSH2 | na | 19838 | -0.012 | -0.2205 | No |
| 158 | CASP9 | na | 19881 | -0.012 | -0.2214 | No |
| 159 | STAT1 | na | 19966 | -0.013 | -0.2235 | No |
| 160 | SMAD3 | na | 19999 | -0.013 | -0.2240 | No |
| 161 | FGF11 | na | 20046 | -0.013 | -0.2249 | No |
| 162 | TCF7L2 | na | 20073 | -0.014 | -0.2253 | No |
| 163 | CYCS | na | 20083 | -0.014 | -0.2251 | No |
| 164 | DVL3 | na | 20205 | -0.015 | -0.2282 | No |
| 165 | RALB | na | 20318 | -0.016 | -0.2310 | No |
| 166 | WNT5B | na | 20321 | -0.016 | -0.2306 | No |
| 167 | BAD | na | 20453 | -0.017 | -0.2339 | No |
| 168 | LAMB2 | na | 20547 | -0.018 | -0.2360 | No |
| 169 | DAPK3 | na | 20614 | -0.018 | -0.2374 | No |
| 170 | TFG | na | 20631 | -0.019 | -0.2373 | No |
| 171 | RAC2 | na | 20865 | -0.021 | -0.2435 | No |
| 172 | CDK4 | na | 21131 | -0.023 | -0.2505 | No |
| 173 | EGLN2 | na | 21217 | -0.024 | -0.2522 | No |
| 174 | CDKN2A | na | 21295 | -0.025 | -0.2537 | No |
| 175 | PIAS4 | na | 21348 | -0.025 | -0.2545 | No |
| 176 | PRKCA | na | 21412 | -0.026 | -0.2555 | No |
| 177 | BIRC5 | na | 21430 | -0.026 | -0.2552 | No |
| 178 | ABL1 | na | 21453 | -0.026 | -0.2551 | No |
| 179 | RXRB | na | 21455 | -0.026 | -0.2543 | No |
| 180 | PIAS2 | na | 21491 | -0.027 | -0.2545 | No |
| 181 | APC2 | na | 21518 | -0.027 | -0.2545 | No |
| 182 | CCDC6 | na | 21772 | -0.030 | -0.2609 | No |
| 183 | WNT4 | na | 21797 | -0.030 | -0.2607 | No |
| 184 | WNT3 | na | 21833 | -0.030 | -0.2608 | No |
| 185 | EGFR | na | 22041 | -0.033 | -0.2659 | No |
| 186 | TRAF2 | na | 22056 | -0.033 | -0.2653 | No |
| 187 | CKS1B | na | 22104 | -0.033 | -0.2657 | No |
| 188 | SMO | na | 22291 | -0.035 | -0.2700 | No |
| 189 | GRB2 | na | 22294 | -0.035 | -0.2690 | No |
| 190 | IKBKB | na | 22300 | -0.035 | -0.2681 | No |
| 191 | ARNT2 | na | 22414 | -0.036 | -0.2703 | No |
| 192 | GSK3B | na | 22452 | -0.037 | -0.2703 | No |
| 193 | PIAS3 | na | 22467 | -0.037 | -0.2696 | No |
| 194 | KITLG | na | 22603 | -0.039 | -0.2724 | No |
| 195 | MAPK8 | na | 22620 | -0.039 | -0.2717 | No |
| 196 | CDC42 | na | 22626 | -0.039 | -0.2706 | No |
| 197 | EPAS1 | na | 22638 | -0.039 | -0.2698 | No |
| 198 | MYC | na | 22660 | -0.039 | -0.2692 | No |
| 199 | MAPK1 | na | 22705 | -0.040 | -0.2693 | No |
| 200 | TGFA | na | 22844 | -0.041 | -0.2721 | No |
| 201 | STK36 | na | 22907 | -0.042 | -0.2726 | No |
| 202 | HSP90AB1 | na | 22914 | -0.042 | -0.2715 | No |
| 203 | WNT2 | na | 22923 | -0.042 | -0.2705 | No |
| 204 | CDKN1B | na | 22949 | -0.042 | -0.2700 | No |
| 205 | RARA | na | 22997 | -0.043 | -0.2700 | No |
| 206 | TPM3 | na | 23026 | -0.043 | -0.2695 | No |
| 207 | IKBKG | na | 23063 | -0.044 | -0.2693 | No |
| 208 | MSH6 | na | 23138 | -0.045 | -0.2701 | No |
| 209 | CRK | na | 23147 | -0.045 | -0.2690 | No |
| 210 | PML | na | 23168 | -0.045 | -0.2682 | No |
| 211 | RASSF1 | na | 23202 | -0.046 | -0.2678 | No |
| 212 | FGF1 | na | 23262 | -0.046 | -0.2681 | No |
| 213 | MLH1 | na | 23263 | -0.046 | -0.2667 | No |
| 214 | WNT9B | na | 23327 | -0.047 | -0.2671 | No |
| 215 | GLI3 | na | 23514 | -0.050 | -0.2710 | No |
| 216 | TCF7 | na | 23571 | -0.051 | -0.2711 | No |
| 217 | PPARD | na | 23638 | -0.052 | -0.2715 | No |
| 218 | ITGA3 | na | 24015 | -0.056 | -0.2807 | No |
| 219 | E2F3 | na | 24031 | -0.057 | -0.2795 | No |
| 220 | JUN | na | 24077 | -0.057 | -0.2790 | No |
| 221 | JAK1 | na | 24146 | -0.058 | -0.2793 | No |
| 222 | BMP4 | na | 24168 | -0.058 | -0.2781 | No |
| 223 | HDAC2 | na | 24190 | -0.059 | -0.2770 | No |
| 224 | DVL1 | na | 24203 | -0.059 | -0.2755 | No |
| 225 | EP300 | na | 24292 | -0.060 | -0.2763 | No |
| 226 | PTK2 | na | 24353 | -0.061 | -0.2762 | No |
| 227 | APC | na | 24380 | -0.062 | -0.2751 | No |
| 228 | BIRC3 | na | 24545 | -0.064 | -0.2779 | No |
| 229 | CSF3R | na | 24553 | -0.064 | -0.2762 | No |
| 230 | FAS | na | 24662 | -0.066 | -0.2774 | No |
| 231 | IL6 | na | 24766 | -0.067 | -0.2783 | No |
| 232 | ARAF | na | 24817 | -0.068 | -0.2778 | No |
| 233 | WNT6 | na | 24905 | -0.069 | -0.2782 | No |
| 234 | PIK3CD | na | 24971 | -0.070 | -0.2780 | No |
| 235 | DVL2 | na | 25071 | -0.072 | -0.2787 | No |
| 236 | FGFR2 | na | 25276 | -0.075 | -0.2824 | No |
| 237 | EGLN1 | na | 25375 | -0.077 | -0.2829 | No |
| 238 | FZD9 | na | 25389 | -0.077 | -0.2810 | No |
| 239 | PTGS2 | na | 25427 | -0.077 | -0.2798 | No |
| 240 | STAT3 | na | 25462 | -0.078 | -0.2784 | No |
| 241 | LAMC1 | na | 25472 | -0.078 | -0.2763 | No |
| 242 | ITGAV | na | 25782 | -0.083 | -0.2828 | No |
| 243 | NFKB1 | na | 25842 | -0.084 | -0.2820 | No |
| 244 | WNT7B | na | 26237 | -0.092 | -0.2907 | Yes |
| 245 | CDKN2B | na | 26262 | -0.093 | -0.2886 | Yes |
| 246 | DAPK2 | na | 26278 | -0.093 | -0.2862 | Yes |
| 247 | HSP90B1 | na | 26345 | -0.095 | -0.2853 | Yes |
| 248 | TGFBR1 | na | 26390 | -0.095 | -0.2837 | Yes |
| 249 | BCL2L1 | na | 26569 | -0.099 | -0.2859 | Yes |
| 250 | NFKB2 | na | 26748 | -0.102 | -0.2880 | Yes |
| 251 | ITGA2 | na | 26752 | -0.102 | -0.2850 | Yes |
| 252 | LAMB1 | na | 26781 | -0.103 | -0.2827 | Yes |
| 253 | SLC2A1 | na | 26795 | -0.103 | -0.2800 | Yes |
| 254 | TCF7L1 | na | 26813 | -0.104 | -0.2773 | Yes |
| 255 | STAT5A | na | 26873 | -0.105 | -0.2759 | Yes |
| 256 | CXCL8 | na | 26911 | -0.106 | -0.2738 | Yes |
| 257 | CRKL | na | 27010 | -0.108 | -0.2734 | Yes |
| 258 | NRAS | na | 27014 | -0.108 | -0.2703 | Yes |
| 259 | CTNNA3 | na | 27076 | -0.110 | -0.2687 | Yes |
| 260 | WNT10A | na | 27282 | -0.114 | -0.2713 | Yes |
| 261 | GLI2 | na | 27332 | -0.115 | -0.2692 | Yes |
| 262 | CDK6 | na | 27414 | -0.117 | -0.2681 | Yes |
| 263 | PIK3CG | na | 27455 | -0.118 | -0.2657 | Yes |
| 264 | NFKBIA | na | 27480 | -0.118 | -0.2628 | Yes |
| 265 | BRCA2 | na | 27586 | -0.121 | -0.2622 | Yes |
| 266 | E2F1 | na | 27800 | -0.126 | -0.2646 | Yes |
| 267 | CSF1R | na | 27843 | -0.127 | -0.2620 | Yes |
| 268 | CDK2 | na | 28335 | -0.139 | -0.2721 | Yes |
| 269 | HIF1A | na | 28452 | -0.142 | -0.2712 | Yes |
| 270 | RXRG | na | 28564 | -0.145 | -0.2701 | Yes |
| 271 | RELA | na | 28616 | -0.147 | -0.2672 | Yes |
| 272 | LAMA3 | na | 28652 | -0.148 | -0.2638 | Yes |
| 273 | ITGA6 | na | 28732 | -0.150 | -0.2616 | Yes |
| 274 | TPR | na | 28794 | -0.151 | -0.2588 | Yes |
| 275 | AXIN2 | na | 28854 | -0.153 | -0.2559 | Yes |
| 276 | COL4A4 | na | 28877 | -0.154 | -0.2519 | Yes |
| 277 | RUNX1 | na | 28898 | -0.154 | -0.2479 | Yes |
| 278 | COL4A2 | na | 28922 | -0.155 | -0.2439 | Yes |
| 279 | SHH | na | 28942 | -0.155 | -0.2398 | Yes |
| 280 | ITGB1 | na | 29102 | -0.160 | -0.2396 | Yes |
| 281 | LAMC2 | na | 29103 | -0.160 | -0.2348 | Yes |
| 282 | LAMB3 | na | 29197 | -0.163 | -0.2326 | Yes |
| 283 | MAP2K1 | na | 29246 | -0.164 | -0.2291 | Yes |
| 284 | PGF | na | 29370 | -0.168 | -0.2276 | Yes |
| 285 | MET | na | 29439 | -0.170 | -0.2245 | Yes |
| 286 | RAD51 | na | 29952 | -0.187 | -0.2337 | Yes |
| 287 | VEGFA | na | 30038 | -0.189 | -0.2305 | Yes |
| 288 | TGFB1 | na | 30914 | -0.218 | -0.2494 | Yes |
| 289 | PAX8 | na | 30919 | -0.218 | -0.2430 | Yes |
| 290 | WNT8B | na | 31267 | -0.231 | -0.2461 | Yes |
| 291 | KLK3 | na | 31394 | -0.236 | -0.2427 | Yes |
| 292 | BMP2 | na | 31395 | -0.236 | -0.2356 | Yes |
| 293 | NOS2 | na | 31398 | -0.236 | -0.2285 | Yes |
| 294 | BCL2 | na | 31549 | -0.242 | -0.2256 | Yes |
| 295 | FZD10 | na | 31652 | -0.246 | -0.2212 | Yes |
| 296 | CDKN1A | na | 31724 | -0.250 | -0.2157 | Yes |
| 297 | VEGFC | na | 31739 | -0.251 | -0.2086 | Yes |
| 298 | WNT7A | na | 31957 | -0.261 | -0.2070 | Yes |
| 299 | ZBTB16 | na | 32190 | -0.276 | -0.2055 | Yes |
| 300 | FZD2 | na | 32387 | -0.287 | -0.2026 | Yes |
| 301 | PPARG | na | 32783 | -0.313 | -0.2047 | Yes |
| 302 | LEF1 | na | 32857 | -0.317 | -0.1973 | Yes |
| 303 | TRAF1 | na | 32905 | -0.321 | -0.1890 | Yes |
| 304 | LAMA1 | na | 33178 | -0.343 | -0.1866 | Yes |
| 305 | COL4A6 | na | 33261 | -0.350 | -0.1785 | Yes |
| 306 | CSF2RA | na | 33430 | -0.364 | -0.1724 | Yes |
| 307 | EGLN3 | na | 33519 | -0.372 | -0.1638 | Yes |
| 308 | WNT2B | na | 33588 | -0.378 | -0.1544 | Yes |
| 309 | AKT3 | na | 33656 | -0.385 | -0.1448 | Yes |
| 310 | MMP1 | na | 33729 | -0.395 | -0.1350 | Yes |
| 311 | GLI1 | na | 33749 | -0.397 | -0.1236 | Yes |
| 312 | WNT1 | na | 33810 | -0.404 | -0.1132 | Yes |
| 313 | FZD7 | na | 33835 | -0.408 | -0.1017 | Yes |
| 314 | ETS1 | na | 33844 | -0.409 | -0.0896 | Yes |
| 315 | FGFR1 | na | 33845 | -0.409 | -0.0773 | Yes |
| 316 | NKX3-1 | na | 33856 | -0.411 | -0.0652 | Yes |
| 317 | WNT11 | na | 33881 | -0.415 | -0.0535 | Yes |
| 318 | PDGFB | na | 34011 | -0.431 | -0.0443 | Yes |
| 319 | PRKCB | na | 34153 | -0.452 | -0.0348 | Yes |
| 320 | MITF | na | 34462 | -0.537 | -0.0276 | Yes |
| 321 | MMP9 | na | 34473 | -0.541 | -0.0116 | Yes |
| 322 | FGF4 | na | 34605 | -0.633 | 0.0036 | Yes |
Table: GSEA details [plain text format]

  

Fig 2: KEGG\_PATHWAYS\_IN\_CANCER      
 Blue-Pink O' Gram in the Space of the Analyzed GeneSet

  

Fig 3: KEGG\_PATHWAYS\_IN\_CANCER: Random ES distribution      
 Gene set null distribution of ES for **KEGG\_PATHWAYS\_IN\_CANCER**
